# Unlocking engagement: exploring the drivers of elderly participation in digital backfeeding through community education

**DOI:** 10.3389/fpsyg.2025.1524373

**Published:** 2025-02-06

**Authors:** Sijie Sun

**Affiliations:** Department of Philosophy, Autonomous University of Barcelona, Barcelona, Spain

**Keywords:** aging, digital backfeeding, community education, technology acceptance model, digital skills, age-friendly design

## Abstract

Amid China’s rapid aging and digitalization, elderly individuals face a “digital refugee” dilemma, making community education a vital channel for enhancing their digital participation. To address this, the study investigates how community education influences elderly engagement in digital backfeeding—a process where younger generations assist older adults with digital skills—by examining the interplay of personal, technological, and community factors in promoting technology acceptance. Using an integrated framework of the UTAUT, TTF, and TAM models, the research surveyed 482 elderly participants in community centers in Taiyuan, China, focusing on variables such as task and technology characteristics, social influence, facilitating conditions, perceived usefulness, ease of use, and technology anxiety. Analysis through AMOS and SPSS Process macro revealed that task characteristics significantly enhance engagement willingness, although technology characteristics introduce certain challenges. Furthermore, social influence and facilitating conditions were found to positively affect willingness and behavior, mediated by perceived usefulness, ease of use, and technology anxiety. Notably, participatory digital skills exhibited a stronger moderating effect on engagement willingness compared to receptive skills. These findings underscore the pivotal role of community education in fostering digital inclusion among the elderly. Practical recommendations include simplifying technology interfaces, creating supportive environments, and prioritizing participatory skills development to enhance technology acceptance, offering valuable insights for the design of age-friendly digital tools that bridge the digital divide.

## 1 Introduction

Currently, China faces the significant challenge of an aging population. With the aging rate surpassing 14.7%, the nation is on the verge of entering a deeply aged society (National Bureau of Statistics of China, 2023; UNPD, 2024). Projections from the United Nations Population Development Report estimate that by 2050, China’s aging rate will reach 30%, with 370 million elderly individuals accounting for 23.8% of the global elderly population (Zhang et al., 2024). As the country with the largest aging population in the world, China encounters pressing challenges in eldercare, including funding, care models, and education for the elderly. Addressing the aging issue has become not only a central concern in China’s socio-economic development but also a long-term fundamental national priority (Xia et al., 2024; Zhang et al., 2024).

Amid this demographic shift, the simultaneous digitalization of society has brought an additional layer of complexity. The rapid development and adoption of digital technologies have ushered China into a new digital era. According to the 53rd Statistical Report on Internet Development in China, as of December 2023, the number of internet users in China has reached 1.092 billion, with an internet penetration rate of 71.6%. This makes China the largest digital society globally. While digitalization has transformed the ways individuals access and share information, it has also fundamentally reshaped public services and social interactions, gradually reducing the constraints of physical space (Emmert-Streib, 2021; Mossberger et al., 2007; Li and Sun, 2024). However, this transition has created significant challenges for older adults, who often experience declines in cognitive abilities and technological adaptability, leaving many feeling overwhelmed and marginalized. The resulting phenomenon of older adults as “digital refugees” requires urgent attention and innovative solutions.

One such solution is digital backfeeding, which has emerged as a promising approach to bridge the digital divide for older adults. This educational process involves younger generations providing support to elders in accessing, using, and mastering digital technologies (Chen et al., 2023; Junxun et al., 2024). Rooted in the concept of “cultural backfeeding,” digital backfeeding emphasizes intergenerational learning and collaboration (Lu et al., 2022). Beyond helping older adults navigate the digital world, this process fosters stronger family relationships and broader societal digital inclusion (Damant et al., 2017; Junxun et al., 2024; Lu et al., 2022). Encouraging active participation in digital backfeeding is essential to overcoming the resistance many older adults feel toward new technologies, improving their willingness to engage with digital tools.

Community education plays a pivotal role in addressing these challenges by serving as a structured platform for lifelong learning. It creates social networks and learning communities tailored to the needs of older adults (Philips et al., 2001). By incorporating digital technologies into its curriculum, community education can effectively break down the psychological and cognitive barriers older adults face, enabling them to develop a more positive mindset toward technology (Hodge et al., 2017). Within these learning environments, older adults not only gain access to necessary devices and instructional support but also benefit from peer interactions, which enhance their motivation and willingness to engage with digital tools (Rahi et al., 2019; Marston and Van Hoof, 2019; Venkatesh et al., 2011). Furthermore, the level of digital participation among older adults is closely tied to the digital skills they possess, which influence their confidence and adaptability (Yang and Jang, 2024).

To address the gaps in existing approaches, this paper investigates how various aspects of community education influence older adults’ willingness to participate in digital backfeeding. By examining the mediating roles of technological and psychological factors, it aims to inform the design of effective digital backfeeding tools, such as interactive games that combine learning, fun, and problem-solving elements. These tools can serve as a pathway to increasing digital engagement among older adults, ultimately helping them transition from “digital refugees” to active participants in the digital age.

### 1.1 Current research on digital backfeeding

Digital backfeeding, a framework aimed at supporting older adults in adapting to digital life, has gained considerable attention, particularly in family and community settings. However, existing digital backfeeding initiatives often lack a formal structure, relying heavily on *ad hoc* and temporary assistance from family members or community volunteers. This informal approach, characterized by its short-term focus, limits the sustainability and effectiveness of efforts to foster long-term digital literacy among older adults (Mannheim et al., 2019; Wang and Zhang, 2024).

In contrast, community education offers a more systematic and sustainable framework for advancing digital inclusion. By providing structured access to teaching resources, peer support, and interactive learning environments, community education addresses many of the limitations inherent in informal support systems. For instance, tools such as puzzle games have demonstrated effectiveness in enhancing digital skills and fostering engagement by offering immediate feedback and promoting a sense of accomplishment (Punchoojit and Hongwarittorrn, 2017). Furthermore, intergenerational interactions within community settings not only facilitate the acquisition of digital competencies but also contribute to strengthening social connections and mitigating generational divides, thereby addressing broader issues of digital exclusion (Bailey and Ngwenyama, 2010). Despite these advancements, research has yet to comprehensively investigate how specific community-related factors—such as social influence and facilitating conditions—impact older adults’ willingness to engage in digital backfeeding. This study seeks to address these gaps by analyzing how key aspects of community education contribute to promoting active participation and better integration of older adults into the digital era.

### 1.2 Personal factor: task characteristics

Task characteristics are defined as “the actions performed by an individual to transform inputs into outputs” (Goodhue and Thompson, 1995; Rahi et al., 2021). In the context of older adults engaging in digital backfeeding, task characteristics encompass the specific activities required to effectively utilize digital technologies and products for information processing (Junxun, 2024). Drawing on the Task-Technology Fit (TTF) model, task characteristics are critical determinants of individuals’ intentions to adopt technology, as the alignment between technology and user needs significantly enhances task efficiency and usability (Wan et al., 2020; Wu and Chen, 2017). Older adults increasingly depend on digital technologies for a variety of tasks, such as scheduling medical appointments, managing finances, and maintaining social connections (Carroll et al., 2020; Thongsri et al., 2018). These applications underscore the importance of functionality and usability in influencing technology adoption (Damant et al., 2017; Sims et al., 2017). For instance, a health-monitoring application designed with simplified navigation, step-by-step guidance, and real-time feedback can encourage older adults to adopt it consistently, addressing both functional requirements and usability concerns. However, challenges such as cognitive decline and limited digital literacy necessitate the development of tailored interventions within community education settings to support technology adoption (Pigeon et al., 2021).

Community education serves as a key enabler in addressing these barriers, operating through two primary pathways: explicit and implicit influences. Explicit influences involve direct instruction from educators, who train older adults to use digital tools for practical problem-solving, such as navigating e-commerce platforms for daily necessities. Implicit influences, on the other hand, stem from peer interactions and educator demonstrations, which shape perceptions of technology’s value, fostering confidence and acceptance (Hodge et al., 2017; Yang and Jang, 2024). These processes interact with mediating factors, such as perceived usefulness and ease of use, and moderating factors, such as digital skills, to enhance older adults’ participation in digital backfeeding. By addressing these interrelated factors, community education provides a supportive environment that mitigates barriers to technology adoption. For example, an interactive workshop on mobile banking, complemented by peer encouragement and simplified training resources, can simultaneously enhance task-specific skills and reduce technological anxiety. Consequently, task characteristics within community education play a pivotal role in promoting older adults’ willingness to participate in digital backfeeding and adopt technological products. Based on this, this study proposes hypothesis 1:

*H1*: Task characteristics have a positive effect on older adults’ (a) willingness to participate in digital backfeeding and (b) technology product usage behavior.

### 1.3 Technological factor: technology characteristics

Technology characteristics refer to “the technology individuals use to perform tasks” (Abdekhoda et al., 2022; Goodhue and Thompson, 1995). For older adults participating in digital backfeeding, these characteristics encompass the tools and features they need to interact effectively with digital content and derive benefits from it. However, as adopting and adapting to new technology often requires more physical effort and cognitive adaptation, older adults tend to perceive technology as more complex compared to younger individuals. This complexity demands higher levels of both physical and mental engagement (Campens et al., 2024; Sims et al., 2017).

Within community education, the perceived difficulty or ease of using technology significantly influences the effort that older adults invest in learning and using these tools. Explicit influence refers to the direct perception of difficulty in mastering new technologies, while implicit influence involves the challenges encountered during the learning process, such as technical problems faced by teachers or peers and the ease with which these issues are resolved (Hodge et al., 2017). For example, if older adults struggle to navigate a complicated online shopping platform, their frustration may hinder their willingness to engage in digital backfeeding. Thus, technology’ s complexity can act as a barrier to participation in digital backfeeding. Therefore, this paper proposes the following hypotheses:

*H2*: Technological characteristics have a negative effect on older adults’ (a) willingness to participate in digital backfeeding and (b) technology product usage behavior.

### 1.4 Community factor: social influence and facilitating conditions

Community factors, according to the widely used UTAUT model for explaining and predicting how users accept and use new technology, identify two peripheral influences that affect technology usage: social influence and facilitating conditions.

#### 1.4.1 Social influence

Social influence refers to the extent to which individuals perceive pressure from their social environment to adopt particular technologies (Lu and Tsai-Lin, 2024; Hsu and Peng, 2022). For older adults, the pressure to engage with technology is not solely driven by the desire to follow trends but is largely motivated by the need to remain connected in a society where digital tools are becoming increasingly ubiquitous. In community settings, older adults may feel an implicit expectation to use smartphones for activities such as participating in group chats or accessing online services, thereby contributing to the broader social demand for digital engagement. This pressure may stem from a variety of sources, including peers, educators, and even younger generations who are keen to assist in bridging the digital divide.

It is important to recognize that social influence does not solely drive individual technology adoption but also plays a crucial role in fostering intergenerational relationships. When older adults observe their peers or educators actively engaging with digital technologies, they are more likely to be encouraged to do the same, thereby reducing feelings of intimidation. This positive feedback loop reinforces engagement, with increased social participation enhancing the willingness to adopt new technologies. Furthermore, social influence is often linked to the perception of social norms, where older adults may feel a moral or social obligation to embrace technology in order to maintain family cohesion, particularly with younger members of their social network (Harfitt, 2018; Garbrah et al., 2020). Given this framework, it is hypothesized that:

*H3*: Social influence has a positive effect on older adults’ (a) willingness to participate in digital backfeeding and (b) technology product usage behavior.

#### 1.4.2 Facilitating conditions

Facilitating conditions refer to the resources and support available to individuals that enable them to adopt and effectively utilize technology (Chen and Chan, 2014). While previous research has primarily focused on technical support, this study underscores the significance of the community environment in providing both practical and emotional assistance to older adults as they navigate technology adoption. Facilitating conditions in the context of community education encompass not only the availability of accessible devices and platforms but also the instructional support provided by educators and peers throughout the learning process.

**Figure 1:**
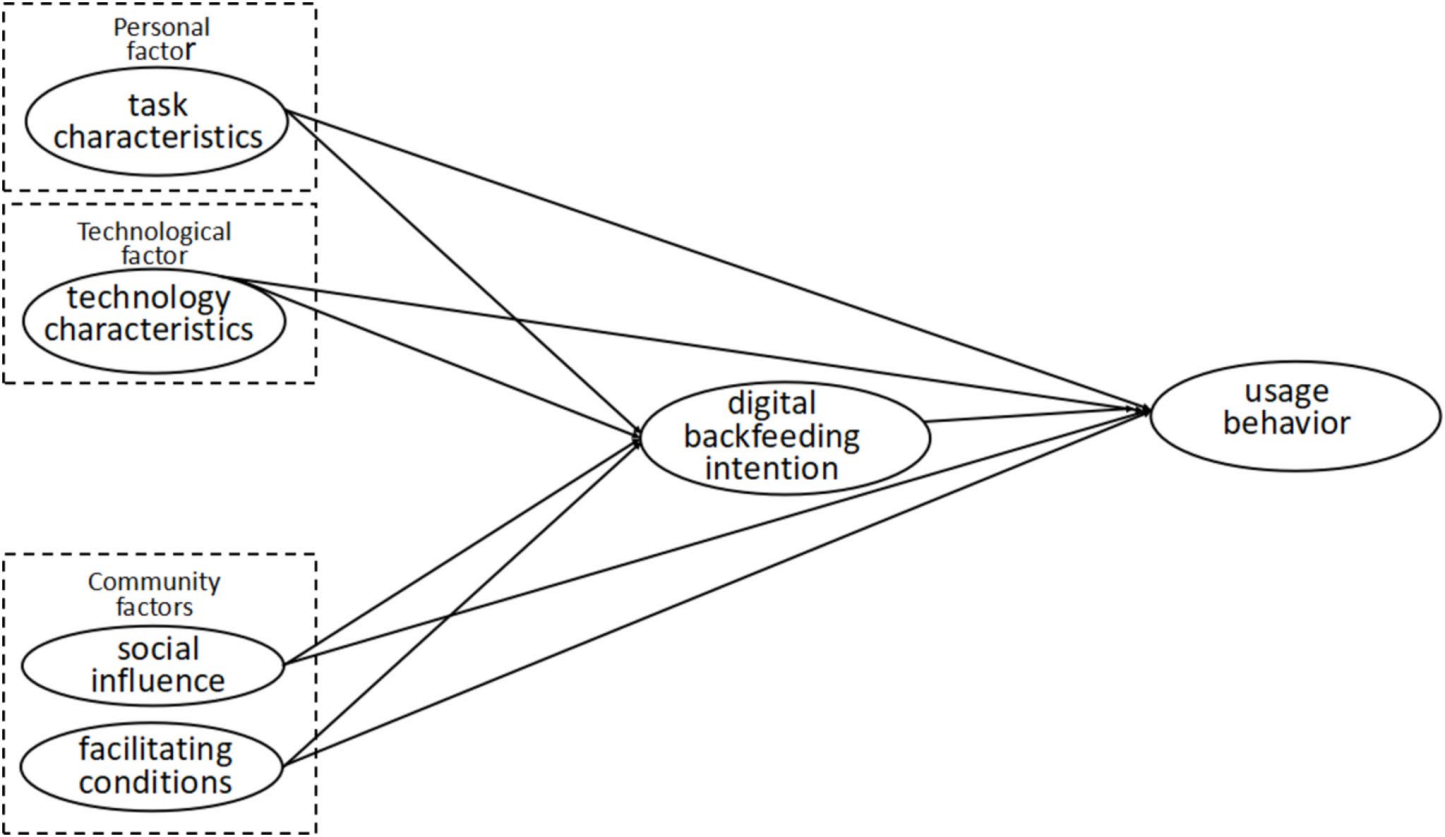
Main research model.

A key component of facilitating conditions is the design of the technology itself. Older adults are more likely to engage with digital tools when these tools are designed with simplicity and accessibility in mind. Technologies featuring large, readable fonts and intuitive navigation help reduce the barriers to usage, while community-driven support structures, such as peer mentoring or instructor-led workshops, provide additional reassurance and guidance throughout the learning process (Figure 1).

Moreover, facilitating conditions extend beyond the mere provision of tools and resources. They also involve the creation of a supportive learning environment in which older adults feel empowered to explore technology without fear of failure. A positive, non-judgmental atmosphere within community education settings can alleviate technological anxiety, which in turn promotes sustained engagement with digital backfeeding. By ensuring a comprehensive support system—comprising both technical assistance and social encouragement—older adults are better positioned to overcome challenges and integrate technology into their daily routines. Based on these considerations, it is hypothesized that:

*H4*: Facilitating conditions have a positive impact on older adults’ (a) willingness to participate in digital backfeeding and (b) technology product usage behavior.

### 1.5 The mediating role of perceived usefulness and perceived ease of use

The Technology Acceptance Model (TAM), introduced by Fred D. Davis in 1986, posits that an individual’s use of an information system is determined by behavioral intentions, which are influenced by two primary factors: perceived usefulness and perceived ease of use. Perceived usefulness refers to the extent to which an individual believes that using a particular system will improve performance, while perceived ease of use pertains to the belief that using the system will require minimal effort (Abdullah et al., 2016; Kalayou et al., 2020). These belief factors collectively shape attitudes toward behavioral intentions, which in turn drive actual usage behavior. While the TAM has been extensively applied, its specific implications for older adults participating in community education remain underexplored. This study aims to expand its application by examining the mediating role of these factors in digital backfeeding.

Within the context of community education, older adults’ perceptions of usefulness and ease of use are influenced by both technological design and the social and instructional environment, which uniquely shape their acceptance of technology. Task characteristics, technological characteristics, and social influence are critical drivers of perceived usefulness. For instance, task characteristics highlight how technology enables older adults to efficiently manage activities like scheduling medical appointments or maintaining social connections, enhancing their sense of control and independence (Oliveira et al., 2014). Technological characteristics, such as user-friendly health monitoring apps, not only streamline task completion but also foster a perception of utility by aligning with the specific needs of older users (Damant et al., 2017; Philips et al., 2001; Wilson et al., 2023). Furthermore, observing peers successfully engaging with technology can amplify social influence, creating a sense of collective utility and reinforcing the belief that adopting digital tools is beneficial.

This interaction suggests that perceived usefulness acts as a crucial bridge between the characteristics of technology and its broader acceptance. Therefore, the following hypothesis is proposed:

*H5*: Perceived usefulness mediates the effect of (a) task characteristics, (b) technological characteristics, and (c) social influence on the willingness of older adults to participate in digital backfeeding.

Conversely, technological characteristics may introduce barriers to perceived ease of use. Complex interfaces or non-intuitive features can deter older adults, particularly those with limited digital experience, from engaging with new tools, as they perceive these features to require excessive cognitive or physical effort (Guner and Acarturk, 2020). However, facilitating conditions can mitigate these barriers. Factors such as clear instructions, simplified interfaces, and consistent technical support help older adults overcome initial challenges, reinforcing their confidence and comfort in using technology. For example, integrating easy-to-understand visual guides or holding regular community workshops can help demystify technology, making it more approachable. Importantly, these facilitating conditions also address the psychological barriers older adults face, such as anxiety and fear of failure, by creating a supportive and non-judgmental learning environment (Barnard et al., 2013; Venkatesh et al., 2008). Given these dynamics, the following hypothesis is proposed:

*H6*: Perceived ease of use mediates the effect of (a) technological characteristics and (b) facilitating conditions on the willingness of older adults to participate in digital backfeeding.

### 1.6 The mediating role of perceived technological anxiety

Perceived technological anxiety refers to the apprehension or discomfort experienced by individuals when interacting with technology, including concerns about their ability to adapt to rapid technological advancements (Lee et al., 2021; Maduku et al., 2023). This anxiety is particularly pronounced among older adults, who may feel uncertain about their capability to navigate digital tools and platforms. Within the context of community education, this phenomenon is further influenced by group pressure, where individuals are subject to the expectations and behaviors of the majority or influential members of their social environment (Asch, 2016). Social influence in communities often exerts dual effects: while observing peers and educators adeptly using technology may inspire older adults to engage, it can simultaneously heighten their anxiety if they perceive themselves as less competent or fear negative judgments. The implicit expectation within communities that older adults must adopt digital tools to remain socially integrated adds further complexity to their acceptance of technology, often amplifying feelings of inadequacy and apprehension.

The interplay between social influence and technological anxiety reveals a nuanced mechanism where group dynamics can either facilitate or inhibit digital adoption. Communities that foster supportive and non-judgmental environments, emphasizing encouragement and gradual learning, are more likely to mitigate technological anxiety, thereby enhancing older adults’ willingness to engage with digital tools. Conversely, settings that prioritize rapid adaptation or peer comparison may intensify anxiety, creating psychological barriers to participation. This dynamic underscores the critical importance of tailoring community-based interventions to address both the emotional and cognitive needs of older adults. Strategies such as one-on-one mentoring, tailored instructional pacing, and consistent reassurance can help alleviate anxiety, promoting self-efficacy and enabling older adults to perceive technology adoption as an attainable goal. Recognizing technological anxiety as a mediating factor provides valuable insight into the mechanisms through which social influence operates, highlighting the need for nuanced approaches to support digital inclusion in aging populations.

Based on this framework, the following hypothesis is proposed:

*H7*: Perceived technological anxiety mediates the effect of social influence on the willingness of older adults to participate in digital backfeeding.

### 1.7 The moderating role of digital skills

As technology becomes increasingly pervasive in society, the digital skills of older adults are continuously evolving and can be categorized into two distinct types: receptive skills and participatory skills (Lee et al., 2011; Yang and Jang, 2024). Receptive skills encompass the ability to interpret, search for, and evaluate digital content, whereas participatory skills involve higher-level activities such as communication, content creation, participation, and sharing within a digital environment (Bartomeu and Ventura, 2021). These two skill types represent different levels of digital engagement, with participatory skills implying a deeper integration of technology into daily life. While receptive skills enable older adults to absorb and process information, participatory skills empower them to actively contribute to digital interactions, express their preferences, and showcase their competencies (Damant et al., 2017; Sims et al., 2017; Wilson et al., 2023).

**Figure 2:**
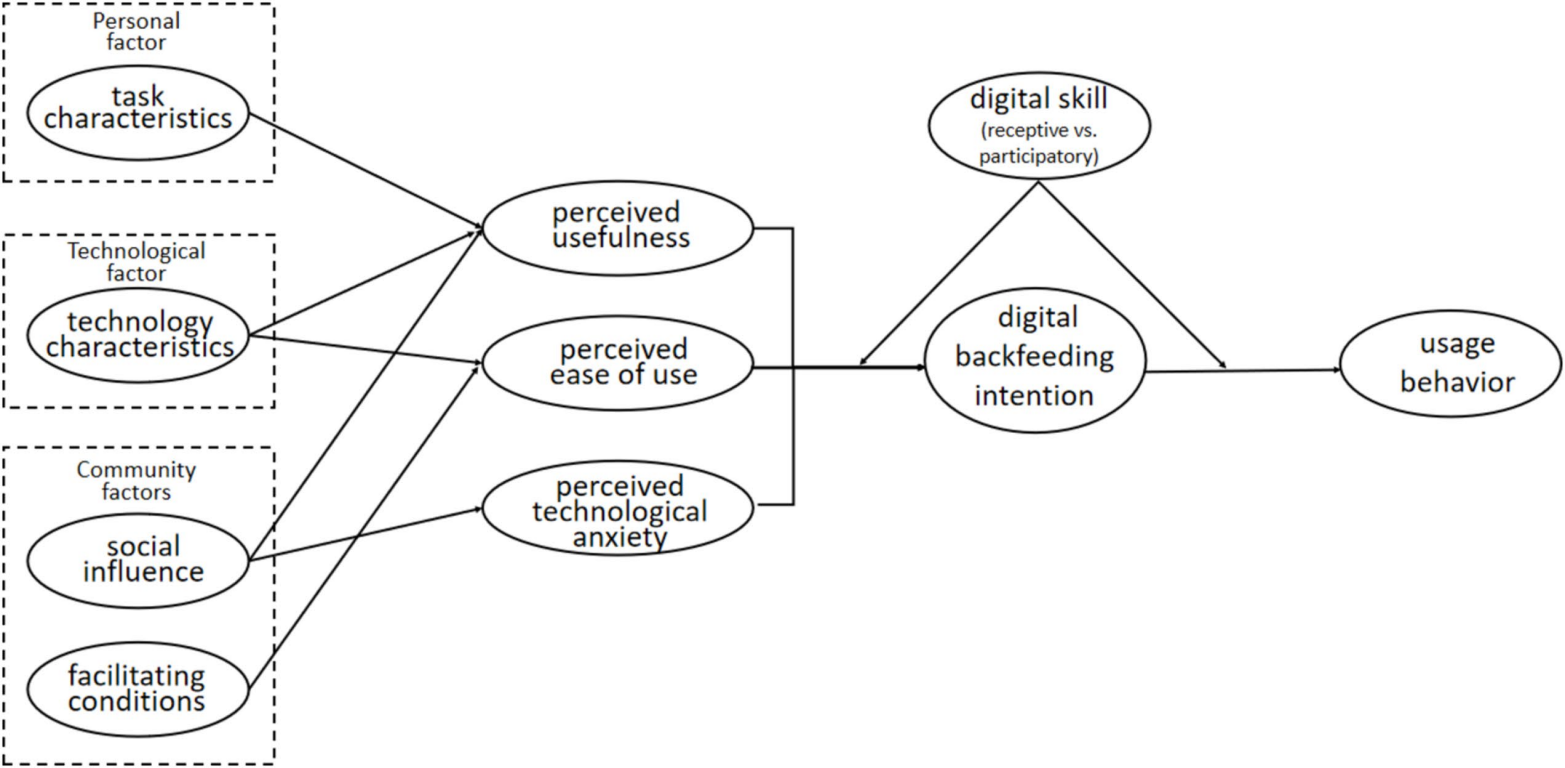
Mediation and moderation model.

For instance, an older adult with strong receptive skills may use a health app to access information about medication schedules or view exercise tutorials, demonstrating their ability to retrieve and evaluate digital content. In contrast, an individual with participatory skills might actively use the same app to share exercise progress with peers, engage in group discussions about health topics, or even customize their digital profile. This level of engagement reflects a proactive approach to digital technology, fostering deeper connections within their community and enhancing their overall digital literacy. Older adults with participatory skills are better positioned to contribute to and benefit from digital backfeeding processes, as they exhibit greater confidence and willingness to explore new technological products and services (Figure 2).

Given the distinction between these skill levels, it is anticipated that participatory skills will amplify the effects of various influencing factors, such as perceived usefulness, social influence, and facilitating conditions, on digital backfeeding engagement (Wu et al., 2021; Vanduhe et al., 2020). Older adults with higher participatory skills are more likely to demonstrate enthusiasm for digital backfeeding and actively use technological products compared to those with only receptive skills. This moderating effect underscores the importance of tailoring educational interventions to not only build basic receptive skills but also cultivate participatory competencies, as they have a stronger influence on digital inclusion and engagement. Based on these considerations, the following hypothesis is proposed:

*H8*: Digital skills (receptive vs. participatory) moderate the effects on older adults’ (a) willingness to participate in digital backfeeding and (b) technology product usage behavior. Older adults with participatory skills exhibit greater willingness and engagement in digital backfeeding and technology usage compared to those with receptive skills.

## 2 Method

### 2.1 Participants

The main study recruited elderly participants from two retirement communities in Taiyuan, Shanxi Province, China, between January 20 and February 20, 2024 for the formal survey. Participants were selected through a convenience sampling method, which ensured that the study focused on a specific elderly population within these communities. While convenience sampling has certain limitations, particularly in terms of representativeness, it was highly suitable for this study as it provided easy access to the target group. Additionally, the selected communities offered a degree of diversity, ensuring that the sample was reasonably representative and the findings valid.

The demographic characteristics of respondents in Table 1 show that 53.1% of the participants in the study were male, and 46.9% were female. 60.0% of the respondents were aged between 65 and 69 years old. The majority had either junior high school (45.2%) or high school (32.4%) education levels. Approximately 15 participants were identified as illiterate, and they received assistance from trained researchers or community volunteers to complete the questionnaire. Regarding health conditions, participants were generally healthy and capable of engaging in community activities. However, some participants reported chronic conditions, such as hypertension or diabetes, which are common among the elderly. No participants with severe neurological disorders, such as advanced dementia or recent strokes, were included in the study. Mild cognitive impairments were not explicitly excluded, as these reflect the general elderly population. To ensure safety, informed consent was obtained, personalized assistance was provided during the survey, and basic medical support was available on-site to address any emergencies.

**Table 1:** Demographics of respondents.

Variable	Description	Frequency	Percentage
Gender	Male	256	53.1%
	Female	226	46.9%
Age	60–64	62	12.9%
	65–69	289	60.0%
	70–74	97	20.1%
	More than 75	34	7.0%
Educational qualification	Elementary school or below	63	13.1%
	Junior high school	218	45.2%
	High school	156	32.4%
	Bachelor or above	45	9.3%

### 2.2 Tests administered

To ensure the validity of all measures, the measurement items for the variables in the proposed model were developed based on previous studies. The structured questionnaire was professionally translated into Chinese for use in the survey. The survey consists of two parts: Part I includes demographic information such as the age, gender, and education level of the respondents. Part II contains questions related to different constructs proposed in the research model (as shown in Table 2), with all items rated on a 5-point Likert scale ranging from 1 (“strongly disagree”) to 5 (“strongly agree”).

**Table 2:** Summary of variable with measurement items.

Variable	Items	Sources
Task characteristics (TaC)	I need to use technological products to bring convenience to my life.	Zhou et al. (2010)
	I need to use technological products to improve my efficiency.	
	I need to use technological products to enhance the quality of my life.	
	I need to use technological products so that I can handle things in life more quickly.	
Technology characteristics (TeC)	Technological products can provide real-time service.	Zhou et al. (2010) and Wang et al. (2020)
	Technological products can provide reliable service.	
	I feel that using technological products in daily life is easy	
	I believe that learning to use technological products is relatively easy for me	
Social Influence (SI)	In my social circle, people who are important to me think that I should use technological products.	Samar et al. (2017)
	In my social circle, technological products are considered important for daily life.	
	In my social circle, people whose opinions I value prefer that I use technological products.	
Facilitating Condition (FC)	There is always a specific person available to help me with learning to use technological products.	Abd Ghani et al. (2017) and Venkatesh et al. (2003)
	For learning to use technological products, I have the necessary technical resources.	
	For learning to use technological products, I possess the knowledge needed during the learning process.	
Perceived usefulness (PU)	Using technological products can bring many benefits to my life.	Nissinen et al. (2024) and Rahi et al. (2019)
	Using technological products allows me to manage my time better.	
	Using technological products can help me connect with others more quickly.	
	Using technological products enables me to quickly access the necessary information in my life.	
	Using technological products can make my life easier.	
Perceived ease of use (PEU)	For me, using technological products is easy.	Rahi et al. (2019) and Wu and Chen (2017)
	I can use technological products flexibly according to my needs.	
	The methods for using technological products are clear.	
	I can easily find guides on how to use technological products.	
	Using technological products is not difficult for me.	
Perceived technological anxiety (PTA)	When people around me use technological products, I feel I must learn to avoid falling behind.	Santor et al. (2000)
	When everyone uses technological products, I find it hard to resist, even if it makes me uncomfortable.	
	If everyone around me uses technological products, I feel stressed and uneasy.	
Willingness to use (WU)	I am very satisfied with handling things using technological products.	Ng et al. (2024)
	I am willing to use technological products to handle matters in my daily life.	
	I find that the various functions within technological products are well integrated, making them convenient to use.	
	When I have tasks to handle, I prefer using technological products over traditional methods.	
Usage behavior (UB)	I use technological products to communicate with friends and family.	Choudhury et al. (2022)
	I use technological products to search for information I need.	
	I use technological products to manage my health issues.	
	I use technological products to solve problems in my daily life.	
	I use technological products to learn and develop myself.	
Digital skill-receptive skill (RS)	I am able to select useful information on the Internet	Van Deursen et al. (2014) and Yang and Jang (2024)
	I can assess how reliable the information I find on the Internet	
	I carefully consider the information I find online	
	I am confident in selecting search results	
	I can correctly understand the information on the Internet	
Digital skill-participatory skill (PS)	I am able to upload photos and videos on the Internet	Koc and Barut (2016) and Yang and Jang (2024)
	I am able to participate in online discussions on social agendas	
	I am good at sharing digital media contents and messages on the Internet	
	I can make contribution or comments to media contents shared by others	
	I can make new friends on the Internet	

### 2.3 Procedure

To test the validity and appropriateness of the survey, a pre-test was conducted on a sample of 30 retired workers from a community in Shanxi Province, China before the formal survey. The backfeeding from this pre-test was used to review and adjust the questionnaire based on specific conditions. The sample had a balanced gender distribution, with an equal number of male and female participants, and ages ranged from 60 to 75 years, with varying levels of education. Based on the feedback, the questionnaire was modified by simplifying complex wording while keeping the original items unchanged, and increasing the font size to enhance accessibility for older participants.

The questionnaire was presented in paper form, and participants were provided assistance from researchers and community volunteers when needed. For illiterate participants, volunteers offered full explanations and assistance to ensure the questionnaire was completed accurately.

The main study was conducted from January 20 to February 20, 2024. The survey was conducted in compliance with Chinese laws and ethical standards, and all participants signed informed consent forms. During the survey process, professional researchers and community volunteers were assigned to provide support to older participants. This support included assisting them in reading and completing the questionnaire and offering clarification and guidance on any questions they found confusing, ensuring that the responses were accurately completed and reflected their true intentions. They were also informed of their right to withdraw from the study at any time without any obligation. All sections were presented in the same order to maintain consistency and comparability across responses. Participants began with general demographic questions, followed by sections on questions related to different constructs proposed in the research model. This fixed order was chosen to ensure a logical flow and to help participants gradually engage with the survey content, starting with simpler and less sensitive questions before moving to more specific or reflective topics.

To further mitigate potential social desirability bias, several methodological measures were implemented. First, participants were assured of complete anonymity, and questionnaires were designed to exclude any identifiable information, reducing the likelihood of socially influenced responses. Second, the wording of questions was carefully crafted to maintain neutrality, avoiding language that might suggest a “strongly agree” or socially acceptable answer. Indirect questioning techniques were also employed for sensitive topics, framing questions in ways that allowed participants to express opinions without direct self-disclosure. Additionally, data collection personnel were trained to maintain a non-judgmental environment and avoid any verbal or non-verbal cues that could influence participants’ answers. These measures, combined with a standardized administration process, aimed to foster an atmosphere of trust and authenticity, ensuring that the data collected accurately reflected participants’ experiences and perspectives. A total of 482 valid questionnaires were collected and used for data analysis.

### 2.4 Statistical procedures

Data analysis was conducted in two stages: (1) the reliability and validity of the measurement model were assessed using Cronbach’s alpha, composite reliability (CR), and average variance extracted (AVE), with all values meeting recommended thresholds. Model fit indices, such as CFI, RMSEA, and SRMR, confirmed the adequacy of the measurement model and (2) structural equation modeling (SEM) with AMOS 26.0 was used to evaluate the structural model, testing direct and indirect relationships between variables. Mediating effects of perceived usefulness, ease of use, and technological anxiety were analyzed using SPSS Process macro v4.0 (Model 1, 4), while moderation analyses examined the role of digital skills.

## 3 Results

### 3.1 Measurement model

This study first conducted a descriptive statistical analysis, with the means and standard deviations shown in Table 3. Next, the measurement model was evaluated by examining internal reliability, convergent validity, and discriminant validity. The results showed that Cronbach’s alpha ranged from 0.776 to 0.946, exceeding the recommended threshold of 0.7. Additionally, both the composite reliability (CR) and the average variance extracted (AVE) (see Table 4) were higher than their suggested standard values of 0.7 and 0.5, respectively (Hair et al., 2011), indicating that the model has satisfactory composite reliability and convergent validity. Moreover, the model fit was assessed using several indices, and the results showed satisfactory fit values. The Comparative Fit Index (CFI) was 0.92, the Root Mean Square Error of Approximation (RMSEA) was 0.05, and the Standardized Root Mean Square Residual (SRMR) was 0.04. These fit indices further support the adequacy of the measurement model and its ability to represent the underlying data structure accurately.

**Table 3:** Descriptive statistics.

	*M*	SD	Cronbach’s alpha
TaC	4.260	0.702	0.832
TeC	3.451	0.635	0.857
SI	4.186	0.761	0.814
FC	4.310	0.662	0.793
PU	4.256	0.711	0.877
PEU	4.302	0.613	0.832
PTA	4.193	0.764	0.776
WU	4.255	0.713	0.850
UB	4.299	0.654	0.870
RS	4.280	0.762	0.907
PS	4.286	0.759	0.946

**Table 4:** The measurement model.

Variable	Items	*B*	S.E	C.R.	*p*	CR	AVE
TaC	JXQW4	1				0.830	0.550
	JXQW3	0.977	0.066	14.768	***		
	JXQW2	1.049	0.068	15.406	***		
	JXQW1	1.036	0.069	14.937	***		
TeC	NLQW4	1				0.858	0.602
	NLQW3	0.965	0.054	17.788	***		
	NLQW2	0.892	0.053	16.69	***		
	NLQW1	0.9	0.053	16.897	***		
SI	SQYX3	1					
	SQYX2	0.986	0.066	14.938	***	0.798	0.569
	SQYX1	1.057	0.069	15.28	***		
FC	SHZC3	1					
	SHZC2	0.819	0.058	14.221	***	0.793	0.561
	SHZC1	1.006	0.066	15.195	***		
PU	GZYYU1	1				0.877	0.587
	GZYYU2	0.894	0.052	17.126	***		
	GZYYU3	0.968	0.053	18.382	***		
	GZYYU4	0.861	0.052	16.543	***		
	GZYYU5	0.957	0.053	18.128	***		
PEU	GZYYX1	1				0.831	0.496
	GZYYX2	0.912	0.068	13.41	***		
	GZYYX3	1.069	0.075	14.257	***		
	GZYYX4	0.958	0.07	13.604	***		
	GZYYX5	0.966	0.071	13.626	***		
PTA	QTYL1	1					
	QTYL2	0.954	0.07	13.679	***	0.779	0.541
	QTYL3	0.906	0.07	12.913	***		
WU	SYYY1	1				0.832	0.554
	SYYY2	1.094	0.067	16.414	***		
	SYYY3	0.947	0.065	14.604	***		
	SYYY4	1.018	0.068	15.014	***		
UB	SYXW1	1				0.866	0.564
	SYXW2	0.873	0.054	16.165	***		
	SYXW3	0.917	0.057	16.225	***		
	SYXW4	0.919	0.056	16.347	***		
	SYXW5	0.973	0.057	17.03	***		

In addition, discriminant validity is assessed by the square root of the AVE and the cross-loading matrix. The square root of the AVE of a construct should be greater than its correlation with other constructs in order to achieve satisfactory discriminant validity (Mustafa et al., 2020). Diagonal elements must be greater than the entries in the corresponding columns and rows for discriminant validity to be satisfied. The results shown in Table 5 indicate that all the constructs in this study confirm the discriminant validity of the data.

**Table 5:** Correlation matrix and square root of the AVE.

	AVE	FC	SI	TeC	TaC	PTA	PEU	PU	WU	UB
FC	0.561	**0.749**								
SI	0.569	0.606	**0.754**							
TeC	0.602	−0.160	−0.161	**0.776**						
TaC	0.550	0.565	0.459	−0.106	**0.742**					
PTA	0.541	0.326	0.538	−0.087	0.247	**0.736**				
PEU	0.496	0.610	0.427	−0.303	0.607	0.230	**0.704**			
PU	0.587	0.445	0.587	−0.105	0.514	0.316	0.378	**0.766**		
WU	0.554	0.464	0.502	−0.172	0.463	0.469	0.560	0.557	**0.744**	
UB	0.564	0.270	0.292	−0.100	0.270	0.273	0.326	0.324	0.582	**0.751**

Note: Bold diagonal values are the square root of AVE, showing reliability. Off-diagonal values are correlations; AVE values should exceed correlations for discriminant validity.

### 3.2 Hypothesis testing

To validate the hypotheses of this study, structural equation modeling (SEM) was conducted, and the standardized path coefficients between the structural variables were examined. Firstly, task characteristics had a significant positive effect on willingness to participate in digital backfeeding (*β* = 0.466, se = 0.041, *p* = 0.000) and technology product usage behavior (B = 0.257, se = 0.057, *p* = 0.000). This indicates that an increase in task characteristics can significantly enhance individuals’ willingness to participate in digital backfeeding and increase their use of technology products. Therefore, Hypothesis 1 is supported.

Next, technological characteristics had a significant negative effect on willingness to participate in digital backfeeding (B = −0.140, se = 0.073, *p* = 0.000) and technology product usage behavior (B = −0.058, se = 0.042, *p* = 0.000), thus supporting Hypothesis 2. Additionally, social influence had a significant positive effect on willingness to participate in digital backfeeding (B = 0.334, se = 0.066, *p* = 0.000) and technology product usage behavior (B = 0.238, se = 0.050, *p* = 0.000), and facilitating conditions had a significant positive effect on willingness to participate in digital backfeeding (B = 0.488, se = 0.064, *p* = 0.000) and technology product usage behavior (B = 0.231, se = 0.063, *p* = 0.000). Therefore, Hypotheses 3 and 4 are also supported (see Table 6).

**Table 6:** Structural model.

Path	*B*	S.E.	C.R.	*p*	Comments
WU <−-- TaC	0.466	0.041	5.662	***	Supported
WU <−-- TeC	−0.140	0.073	−4.574	***	Supported
WU <−-- SI	0.334	0.066	9.075	***	Supported
WU <−-- FC	0.488	0.064	5.79	***	Supported
UB <−-- TaC	0.257	0.057	5.973	***	Supported
UB <−-- TeC	−0.058	0.042	−4.857	***	Supported
UB <−-- SI	0.238	0.050	5.731	***	Supported
UB <−-- FC	0.231	0.063	8.324	***	Supported
PU <−-- TaC	0.347	0.061	5.662	***	Supported
PEU <−-- TeC	0.344	0.056	6.151	***	Supported
PTA <−-- SI	0.538	0.059	9.075	***	Supported
PU <−-- SI	0.439	0.057	7.723	***	Supported
PEU <−-- FC	0.328	0.057	5.79	***	Supported
WU <−-- PU	0.284	0.044	6.482	***	Supported
WU <−-- PEU	0.399	0.056	7.112	***	Supported
WU <−-- PTA	0.241	0.043	5.592	***	Supported
UB <−-- WU	0.611	0.059	10.414	***	Supported

*p < 0.05, **p < 0.01, ***p < 0.001.

Additionally, to test Hypothesis 5, 6, 7, the SPSS Process macro Model 4 was used to analyze the mediating effects proposed in the hypotheses. The analysis results showed that perceived usefulness significantly mediated the effect of task characteristics (*B* = 0.098, se = 0.045, *p* = 0.000), technological characteristics (*B* = 0.137, se = 0.057, *p* = 0.000), and social influence (*B* = 0.125, se = 0.048, *p* = 0.000) on the willingness of older adults to participate in digital backfeeding. Therefore, Hypotheses H5(a), H5(b), and H5(c) were all supported.

Secondly, perceived ease of use significantly mediated the effect of technological characteristics (*B* = –0.074, se = 0.027, *p* = 0.000) and facilitating conditions (*B* = 0.131, se = 0.056, *p* = 0.000) on the willingness of older adults to participate in digital backfeeding. Hence, Hypotheses H6(a) and H6(b) were supported.

Lastly, the mediating effect of perceived technological anxiety on the influence of community impact on the willingness of older adults to participate in digital backfeeding (*B* = 0.129, se = 0.050, *p* = 0.000) was also significant. Therefore, Hypothesis 7 was supported (see Table 7).

**Table 7:** Mediating effect.

Path	*B*	SE	Lower	Upper	*p*
ind1:TaC--PU--WU	0.098	0.045	0.045	0.203	0.002
Ind2:TeC--PU--WU	0.137	0.057	0.069	0.263	0.000
Ind3:SI--PU--WU	0.125	0.048	0.069	0.231	0.000
ind4:TeC--PEU--WU	−0.074	0.027	−0.134	−0.04	0.000
ind5:FC--PEU--WU	0.131	0.056	0.065	0.253	0.000
ind6:SI--PTA--WU	0.129	0.050	0.072	0.237	0.000

Finally, to verify Hypothesis 8, this study utilized SPSS Process Macro Model 1 to examine the moderating role of digital skills (receptive vs. participatory skills). Firstly, when digital skills (receptive and participatory) were considered as moderating variables between perceived usefulness and willingness to use, the analysis results indicated that participatory skills exhibited a significant interaction effect (*B* = 0.100, SE = 0.030, *p* = 0.001), whereas receptive skills did not show a significant moderating effect (*B* = 0.031, SE = 0.037, *p* = 0.396). Secondly, when digital skills (receptive and participatory) were considered as moderating variables between perceived ease of use and willingness to use, both receptive skills (*B* = 0.104, SE = 0.043, *p* = 0.015) and participatory skills (*B* = 0.118, SE = 0.031, *p* = 0.000) showed significant interaction effects. Next, when digital skills (receptive and participatory) were considered as moderating variables between perceived technology anxiety and willingness to use, participatory skills exhibited a significant interaction effect (*B* = 0.139, SE = 0.032, *p* = 0.000), while receptive skills did not show a significant moderating effect (*B* = 0.001, SE = 0.035, *p* = 0.975).

Lastly, when digital skills (receptive and participatory) were considered as moderating variables between willingness to use and actual usage behavior, both receptive skills (*B* = 0.106, SE = 0.031, *p* = 0.001) and participatory skills (*B* = 0.192, SE = 0.031, *p* = 0.000) demonstrated significant interaction effects. Therefore, Hypothesis 8 was partially supported (see Table 8).

**Table 8:** Moderating effect.

	coeff	se	*t*	*p*
PU	0.336	0.040	8.351	0.000
RS	0.222	0.036	6.228	0.000
PU*RS	0.031	0.037	0.850	0.396
PS	0.351	0.038	9.132	0.000
PU*PS	0.100	0.030	3.320	0.001
*R^2^*	0.438			
*F*	74.175			
PEU	0.417	0.048	8.622	0.000
RS	0.214	0.036	6.027	0.000
PEU*RS	0.104	0.043	2.442	0.015
PS	0.359	0.037	9.805	0.000
PEU*PS	0.118	0.031	3.842	0.000
*R^2^*	0.446			
*F*	76.623			
PTA	0.273	0.037	7.315	0.000
RS	0.221	0.036	6.048	0.000
PTA*RS	0.001	0.035	0.031	0.975
PS	0.384	0.039	9.790	0.000
PTA*PS	0.139	0.032	4.402	0.000
*R^2^*	0.432			
*F*	72.392			
WU	0.422	0.044	9.567	0.000
RS	0.092	0.040	2.296	0.022
WU*RS	0.106	0.031	3.431	0.001
PS	0.232	0.044	5.274	0.000
WU*PS	0.192	0.031	6.269	0.000
*R^2^*	0.320			
*F*	44.726			

## 4 Discussion

### 4.1 General discussion

This study, set in elderly communities, employs an integrated model combining the Unified Theory of Acceptance and Use of Technology (UTAUT), Task-Technology Fit (TTF), and Technology Acceptance Model (TAM) to explore how community education influences elderly individuals’ willingness to engage in digital backfeeding and their actual use of technological products. The findings reveal several noteworthy insights that align with or diverge from existing literature and offer novel contributions to the field.

First, the positive impact of task characteristics on willingness underscores the practical value of technology in managing daily tasks. This finding is consistent with prior research demonstrating the importance of perceived task-technology alignment in influencing adoption behaviors (Goodhue and Thompson, 1995; Wu and Chen, 2017). However, this study extends the understanding by highlighting the mediating role of perceived usefulness, showing that when older adults perceive technology as practically beneficial, they are more likely to engage with it through structured support provided in community education settings.

Second, the negative effect of technological characteristics on willingness highlights the persistent challenges older adults face in adopting complex digital systems. This result aligns with studies identifying technological complexity as a barrier to adoption (Damant et al., 2017; Guner and Acarturk, 2020). However, this study advances the literature by demonstrating that perceived ease of use mediates this relationship, suggesting that efforts to simplify technology interfaces, combined with training within community education, can alleviate perceived difficulties and enhance willingness to engage.

Third, the study identifies mediating roles for perceived usefulness, ease of use, and technological anxiety. These findings reaffirm the central tenets of TAM (Davis, 1989) while extending them to the specific context of community education. For example, while moderate technological anxiety has been linked to motivation in prior research (Lee et al., 2021), this study provides new evidence that tailored support in community education can transform anxiety into a facilitator for engagement, emphasizing the importance of emotional support in technology adoption.

Finally, the moderating role of digital skills provides a distinctive contribution by differentiating between receptive and participatory skills. Previous research has primarily focused on general digital literacy (Helsper and Eynon, 2013), but this study shows that participatory skills exert a stronger influence on willingness and actual behavior. This highlights the importance of fostering participatory skills in community education programs to empower older adults to actively engage with technology, providing a novel perspective on digital inclusion strategies.

### 4.2 Implications

#### 4.2.1 Theoretical implications

This study makes significant theoretical contributions by advancing the understanding of digital backfeeding within the elderly population. By integrating UTAUT, TTF, and TAM into a unified framework, it not only validates existing constructs but also extends them to a novel context. Unlike previous studies that focus on younger or general populations (Venkatesh et al., 2003; Wang et al., 2020), this research uniquely applies these models to explore the intersection of aging, technology adoption, and community education.

The concept of “digital backfeeding,” emphasized in this study, enriches the literature by conceptualizing it as both an intergenerational learning process and a pathway to digital inclusion. This builds on the work of Damant et al. (2017), who highlighted the importance of intergenerational interactions, and extends it by providing a systematic analysis of how community education structures influence this process. Furthermore, by focusing on culturally localized settings, the study contributes to understanding how community-driven approaches can address challenges specific to older adults, complementing studies that prioritize individualistic learning frameworks (Chen and Chan, 2014).

Additionally, the differentiation between receptive and participatory digital skills introduces a novel theoretical perspective. Previous research often treats digital literacy as a homogeneous construct (van Deursen et al., 2014), while this study demonstrates that participatory skills have a disproportionately greater influence on technology acceptance and usage behaviors. This insight opens new avenues for future research to explore skill-specific interventions, particularly those aimed at empowering older adults to actively use technology.

Finally, this study bridges theoretical models with practical implications for age-friendly design. While earlier studies advocate for simplifying user interfaces (Barnard et al., 2013), this research empirically links these design elements to enhanced engagement and reduced anxiety, mediated through community education. By integrating theoretical insights with actionable recommendations, this study provides a robust foundation for advancing gerontechnology and promoting digital inclusion for aging populations.

#### 4.2.2 Practical implications

At the practical level, the findings of this study offer significant guidance for advancing age-friendly design and promoting digital inclusion among older adults. The positive impact of task characteristics on digital technology adoption underscores the necessity of embedding practical, task-oriented applications into digital education programs for older adults. Addressing specific needs, such as combating loneliness or managing health, through AI-powered voice assistants and smart devices can provide tangible benefits, enhancing perceived usefulness and motivating sustained engagement. For instance, AI systems designed to facilitate meaningful interactions or provide real-time health monitoring not only address practical concerns but also foster emotional connection and trust in technology (Dikken et al., 2020; Handler, 2018).

This study further highlights the negative influence of technological complexity, emphasizing the critical role of usability in technology design. Simplifying user interfaces is imperative; features such as larger, high-contrast buttons, voice navigation, and intuitive workflows can significantly reduce cognitive barriers for older adults. These design considerations should align with principles of universal design, ensuring that technological products accommodate the sensory and motor limitations commonly experienced in aging populations. Integrating step-by-step visual prompts and adaptive interfaces that adjust to individual user capabilities can further enhance accessibility and engagement.

The findings also underscore the importance of social influence and facilitating conditions in fostering digital participation. Community-driven support structures, such as age-friendly technology centers and specialized training programs, serve as vital enablers, equipping older adults with the resources and confidence to adopt new technologies (Sun et al., 2022). Beyond formal training, community-based initiatives—such as mutual assistance programs—can facilitate intergenerational collaboration, leveraging the technical proficiency of younger individuals to assist older adults in navigating digital environments. These initiatives not only bridge the digital divide but also strengthen intergenerational bonds, fostering a more inclusive technological ecosystem.

Finally, the moderating effect of participatory digital skills reveals a critical avenue for intervention. Older adults with stronger participatory skills exhibit higher levels of willingness and engagement, indicating the need for educational programs that go beyond basic digital literacy. Intergenerational activities, such as co-designed digital backfeeding games, can serve as an innovative tool to foster participatory competencies while enhancing social interaction. Game designs should be informed by gerontological principles, incorporating features such as adjustable difficulty levels, culturally relevant content, and cooperative gameplay modes to ensure sustained motivation and enjoyment (Loos et al., 2019). Such interventions not only promote active technology use but also position older adults as contributors to digital ecosystems, transforming their role from passive recipients to active participants (Zhang et al., 2024).

### 4.3 Limitations and future directions

While this study provides valuable insights into the digital engagement of older adults in a specific community, its geographical limitation significantly affects the generalizability of the findings. The sample was drawn from two communities in Taiyuan, Shanxi Province, China, which may not fully represent the diverse socio-economic, cultural, and regional backgrounds of the broader elderly population across China. As a result, the findings may be particularly relevant to similar communities in the region but may not apply to other areas with distinct cultural or economic conditions.

The specific characteristics of this community—such as access to technology, levels of education, and social support structures—could influence how older adults interact with digital tools. Therefore, the results may be skewed by regional factors that do not necessarily reflect the experiences of older adults in urban or rural areas outside of Shanxi, or in different cultural settings. For instance, elderly individuals in more urbanized regions may have greater access to technology and higher digital literacy, potentially leading to more positive attitudes toward technology adoption compared to those in less developed or rural areas.

Additionally, the study did not consider the experiences of illiterate participants, who may encounter unique barriers to digital engagement due to their inability to read or write. Their reliance on external assistance may have introduced variability in the responses, as comprehension and interpretation could differ based on the helper’s input. Illiteracy can significantly hinder individuals’ ability to navigate digital tools and understand written instructions, potentially resulting in an underrepresentation of their challenges and perspectives in the findings.

To address these limitations, future research should strive to expand the sample to include elderly individuals from a broader range of geographical regions, socio-economic backgrounds, cultural contexts, and literacy levels. This would help ensure the findings are more representative and uncover potential differences in digital technology adoption among older adults from diverse settings. Additionally, future studies should examine how cultural factors, societal norms, and literacy challenges influence the willingness and ability of elderly individuals to engage in digital activities. Special attention should also be given to the inclusion of illiterate participants and the “super-aged” elderly population, as their unique barriers and needs in adopting digital technologies remain underexplored. Such efforts could provide a more comprehensive understanding of the challenges and opportunities for achieving digital inclusion across diverse elderly populations.

## Data Availability

The dataset supporting the conclusions of this article is available in the figshare repository: Sun, Sijie (2025). Question - Unlocking Engagement: Exploring the Drivers of Elderly Participation in Digital Backfeeding through Community Education. figshare. Dataset. https://doi.org/10.6084/m9.figshare.28295246.v2.
